# Different mechanisms of mitral regurgitation in hypertrophic cardiomyopathy: A clinical case and literature review

**DOI:** 10.3389/fcvm.2022.1020054

**Published:** 2022-10-28

**Authors:** Michela Molisana, Adelina Selimi, Germana Gizzi, Simone D’Agostino, Umberto Ianni, Vito Maurizio Parato

**Affiliations:** ^1^Cardiology and Cardiac Rehabilitation Unit, Madonna del Soccorso Hospital, San Benedetto del Tronto, Italy; ^2^Cardiology and Arrhythmology Clinic, University Hospital “Umberto I-Lancisi-Salesi”, Ancona, Italy; ^3^Politecnica delle Marche University, School of Medicine, Ancona, Italy

**Keywords:** hypertrophic cardiomyopathy, LVOT obstruction, systolic anterior motion (SAM), chordal rupture, mitral regurgitation, case report, mitral valve flail, review

## Abstract

**Background:**

Abnormalities of the mitral valve (MV) apparatus are typical features of hypertrophic cardiomyopathy (HCM). These abnormalities include leaflet elongation, thick leaflets, displacement of papillary muscle, and systolic anterior motion (SAM) of the MV anterior leaflet. Mitral valve chordal rupture associated with HCM is a rare but serious issue capable of change the clinical apparence and the prognosis of the patient.

**Case summary:**

A 57-year-old lady with a history of diabetes, dyslipidemia, and a previous single episode of atrial fibrillation (treated with pharmacological cardioversion), presented to the Emergency Department for worsening dyspnea (New York Heart Association Classification class IV). A trans-thoracic echocardiogram (TTE) showed a significant, septal, and asymmetric left ventricular hypertrophy (basal anteroseptal wall diastolic thickness of 19 mm) with normal left ventricle systolic function. A SAM of AML was evident together with a left ventricular outflow tract gradient of 56 mmHg at rest, rising to 136 mmHg during the Valsalva maneuver. In addition, there was evidence of moderate to severe mitral regurgitation (MR) with an anteriorly directed jet, not very typical of MR related to SAM. A 2D-3D trans-esophageal echocardiogram (2D-3D TEE) revealed a combined MR mechanism based on PML degenerative prolapse with P2-flail from ruptured chordae with related eccentric anteriorly directed regurgitant jet, together with a second regurgitant posteriorly directed jet, related to SAM of AML. The patient underwent MV repair together with septal myectomy, with a good final outcome.

**Conclusion:**

Pre-operative echocardiography (both TTE and 2D-3D TEE) is an essential tool in order to detect different MV abnormalities in patients with HCM. These types of patients should never be treated by septal reduction alone. Surgical MV repair or replacement, together with septal myectomy, may be the preferred approach.

## Introduction

Hypertrophic cardiomyopathy (HCM) is the most frequent monogenic cardiac disease, with a prevalence of almost 1 in 500 individuals (1–4). In about 70% of patients with HCM, left ventricular (LV) outflow tract (LVOT) obstruction is due to both septal hypertrophy and pressure drop above the aortic valve (AV) leading to drag forces, attracting the anterior mitral leaflet (AML) and frequently causing significant mitral regurgitation (MR) (5, 6). This kind of MR is called “systolic anterior motion (SAM)-dependent” and it is the most frequently related to HCM. Nevertheless, in up to 10–20% of HCM patients, “SAM-independent MR” may occur, due to intrinsic mitral valve (MV) abnormalities (5).

In almost 3% of cases, MR is the consequence of degenerative MV prolapse of the posterior mitral leaflet (PML). Of these, 19% of cases present an associated PML flail due to chordae tendineae rupture (7). The correct understanding of the MV regurgitation mechanism is crucial and leads to the proper surgical treatment of the pathology.

## Case presentation

**FIGURE 1:**
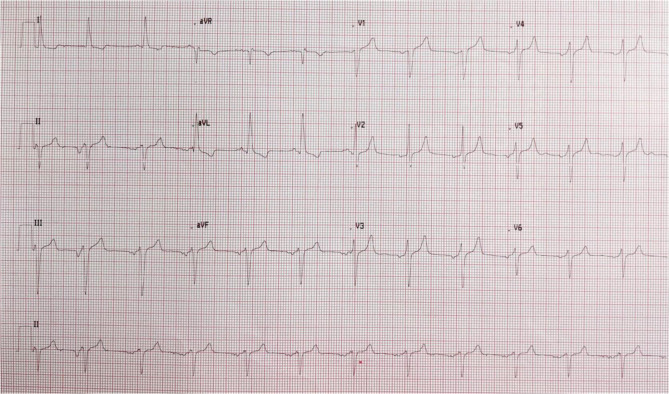
Electrocardiogram: ectopic atrial rhythm, left anterior hemiblock, signs of left ventricular hypertrophy.

A 57-year-old lady with a history of diabetes, thyroid disease, dyslipidemia, and an episode of atrial fibrillation, resolved with pharmacological cardioversion several years before, presented to the Emergency Department for worsening dyspnea (New York Heart Association Classification—NYHA: IV). Her medical therapy included levothyroxine once daily, dapagliflozin 10 mg once daily, metformin 1,000 mg twice daily, rosuvastatin 5 mg, and ezetimibe 10 mg every night. She had no prior heart failure admissions. Other symptoms included orthopnea and paroxysmal nocturnal dyspnea but no chest pain or syncopal episodes. Physical examination showed a pulse rate of 95 bpm, blood pressure of 140/70 mmHg, and 97% of peripheral oxygen saturation. Cardiac auscultation revealed a pansystolic murmur of 3/6 Levine grade over the apex as well as an ejection systolic murmur over the second intercostal space at the left sternal border. There were moderate bibasal lung crepitations and mild ankles edema. Intravenous furosemide was given with resolution of the lungs and ankles edema. Her electrocardiogram showed ectopic atrial rhythm, left anterior hemiblock as well as LV hypertrophy (Figure 1). Laboratory tests showed troponine T of 20 pg/ml (URL of 14 pg/ml), N-terminal pro B-type natriuretic peptide (NT-proBNP) of 582 pg/ml (URL <150 pg/ml).

**FIGURE 2:**
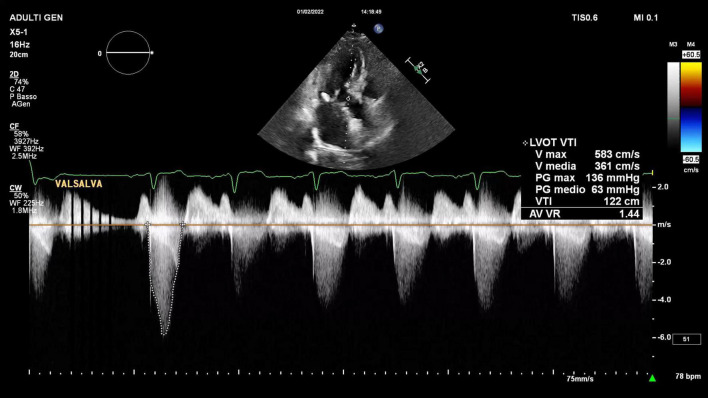
Two-dimensional trans-thoracic echocardiogram continuous wave Doppler representation of LVOT peak gradient at rest and during Valsalva maneuver.

**FIGURE 3:**
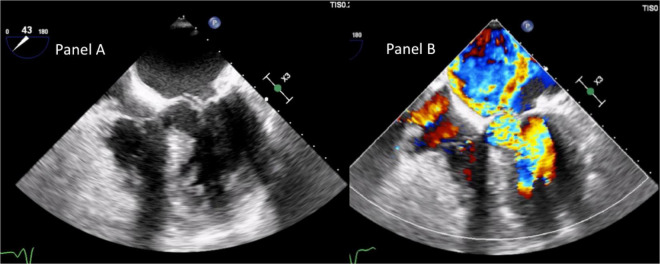
Trans-esophageal echocardiogram mid-esophageal 5-C view demonstrating, in panel **(A)**, basal interventricular septum severe hypertrophy, SAM of AML, chordal rupture, and the related PML-flail. Panel **(B)** shows the two different jets there were at color Doppler. The posteriorly directed jet was due to SAM-related MR. The anteriorly directed jet was related to PML-flail.

A trans-thoracic echocardiogram showed a significant septal, asymmetric LV hypertrophy (basal anteroseptal wall diastolic thickness of 19 mm). Left and right ventricular systolic functions were normal. There was a clear SAM of the AML and an LVOT gradient of 56 mmHg at rest rising to 136 mmHg during the Valsalva maneuver (Supplementary Video 1 and Figure 2). In addition, there was evidence of moderate to severe MR with a significant anteriorly directed jet, not very typical of SAM-related MR. A large calcification at PML-base was noted. 2D-3D trans-esophageal echocardiogram (2D-3D TEE) revealed the true MR mechanism. There was an MR combined mechanism based on: (1) PML degenerative prolapse with P2-flail from ruptured chordae with an eccentric anteriorly directed regurgitant jet and (2) a second posteriorly directed jet, related to SAM of AML (Figures 3A,B and Supplementary Videos 2–4). The pre-operative end-systolic mitral annular diameter was 39 mm while the end-diastolic diameter was 34 mm. There were no signs of infective endocarditis. We added beta-blockers and intravenous diuretics to pre-existing therapy and her clinical condition improved together with a significant LVOT gradient reduction (from 56 to 30 at rest). She underwent coronary-computed tomography angiography that excluded the presence of significant coronary stenoses. She was discharged and, one month later, she underwent MV surgical repair (artificial ring annuloplasty plus artificial chordae implantation) together with septal myectomy. Septal myectomy was performed according to modified Morrow’s procedure. The ruptured chorda was resected during the operation. Regarding annuloplasty, a Carpentier-Edwards Physio II ring N. 32 (Edwards Lifesciences, Irvine, CA, USA) was used. Chordal reconstruction was performed using n. 2 ePTFE Goretex neo-chordae (W.L. Gore & Associates, Flagstaff, AZ, USA). Gore-Tex chords were anchored at the papillary muscle and individually sutured to the free posterior leaflet margin. This intervention shifted the coaptation line posteriorly and contributed to fully resolving AML-SAM. The final outcome was good.

## Discussion

### Mechanisms of mitral regurgitation in hypertrophic cardiomyopathy

#### Systolic anterior motion-dependent mitral regurgitation

Systolic anterior motion-dependent MR is a typical picture of the obstructive form of HCM. It is due to the narrowed LVOT producing a rapid acceleration of blood flow, inducing drag forces to attract the AML during mid and late systole. The result is a SAM of AML worsening LVOT gradient and an eccentric regurgitation jet, directed to the posterolateral wall of the left atrium (5, 6).

Systolic anterior motion is distinguished in three grades of severity:

• Mild: no contact between mitral leaflet and ventricular septum, with a minimum distance of 10 mm.
• Moderate: mitral leaflet-septal contact <30% of systolic time.
• Severe: mitral leaflet-septal contact >30% of the systole duration (6).

The SAM can involve not only the mitral leaflet but also the chordae tendineae but usually, this condition alone is not able to produce a significant LVOT obstruction (6).

#### Systolic anterior motion-independent mitral regurgitation

Intrinsic abnormalities of MV apparatus, with or without the coexisting presence of LVOT obstruction, can lead to different kinds of MR.

##### Leaflet abnormalities

About half of patients with HCM present an abnormal elongation of MV leaflets (5). The first theory explaining this finding combined the presence of anterior malposition of papillary muscles and the drag forces at the LVOT in cases of severe obstruction: this could determine progressive traction on the leaflets, causing elongation (5). Anyway, different published series testified the presence of MV leaflets elongation in patients carrying the HCM-related gene mutations without phenotypic expressions (8, 9), suggesting that sarcomere gene mutations, rather than shear stress or ventricular remodeling, may be responsible for leaflets elongation. In particular, the length of the anterior leaflet in HCM patients is about 34 versus 24 mm in normal hearts, while the posterior leaflet can be longer than 14 mm (10). These characteristics enable the development of MV prolapse and flail. Anyway, despite excessive leaflet elongation and increased leaflet area are present in about 50% of HCM patients (5), analyzing large series of patients, only 3% develop MV prolapse and only 1% mitral chordae rupture with consequent flail (7, 11), suggesting that the mechanism behind these alterations is only partially understood. It seems relevant that, even if the mitral leaflets elongation is very common in HCM and is not related to shear stress, chordal rupture usually appears in patients with severe forms of dynamic obstruction with a significant peak LVOT gradient, dropping after the flail appearance. This may suggest that the drag forces may have a crucial role in inducing the rupture of a sub-valvular apparatus already formed abnormally (11).

##### Sub-valvular abnormalities

Sub-valvular abnormalities usually involve papillary muscles which can be abnormal in position, number, and form in about 50% of patients with HCM.

We may have several possibilities:

a. Hypertrophy of papillary muscle heads.
b. Higher number of papillary muscles (3–4 papillary muscles are present in more than 50% of HCM patients).
c. Anterior and apically displaced papillary muscles. This alteration can shift the MV leaflets anteriorly, leading to chordal and leaflet laxity.
d. Anomalous insertion of one or both heads of the anterolateral papillary muscle directly to the ventricular surface of the AML (5).

##### Mitral valve annulus abnormalities

Changes in size, shape, motion, and angulation of the MV annulus are less common but still possible in presence of LVOT obstruction and deviation of mitral-septal angle (5).

### Mitral valve chordal rupture and mitral flail in hypertrophic cardiomyopathy

#### Etiopathogenesis

Prevalence and pathophysiological mechanisms of MV chordal rupture in HCM are unknown. While a higher prevalence of chordal rupture (5.4%) has been reported in a small surgical series (12), a larger cohort of HCM patients showed that chordal rupture is a rare event in HCM (1%) (11). From a literature search, we found 26 cases of HCM with ruptured chordae tendineae (Table 1) (11–27).

**TABLE 1:** This table summarizes a literature search regarding published cases report in which HCM in association with mitral chordal rupture is described.

References	Sex	Age (years)	AD-HF	Leaflet (scallop) involved	LVOTO prior to chordal rupture	LVOTO after chordal rupture	Treatment	Post-operative LVOTO	Surgical or histological findings
Zhu et al. (12)	M	69	Yes	PML	N/A	No	MVR	N/A	Thickening of AML, calcification of the base of PML. Mucinous degeneration of chordae tendinae
Boissier et al. (11)	F	68	Yes	P3	N/A	Yes (90–100 mmHg)	Morrow + MVr	N/A	MV posterior anulus calcification, no myxomatous changes
Boissier et al. (11)	F	77	Yes	P3	Yes (158 mmHg)	Yes	Morrow + MVr + CABG	N/A	No myxomatous changes
Boissier et al. (11)	M	39	Yes	P2	N/A	Yes (29–64 mmHg)	Morrow + MVr	Yes	No myxomatous changes
Boissier et al. (11)	F	76	Yes	PML	N/A	No	Morrow + MVr	N/A	N/A
Boissier et al. (11)	M	26	Yes	A2	N/A	N/A	Morrow	N/A	N/A
Yamasaki et al. (13)	N/A	N/A	Yes	PML	N/A	Yes	Morrow + MVR	N/A	N/A
Moya Mur et al. (14)	M	61	Yes	PML + AML	N/A	No	MVR	No	N/A
Shibata et al. (15)	F	70	Yes	PML	N/A	Yes (125 mmHg)	Morrow + MVR	No	N/A
Kioka et al. (16)	F	53	Yes	PML	Yes	Yes (75 mmHg)	Morrow + MVr	No	No myxomatous changes
Yeo et al. (17)	M	59	Yes	PML	N/A	Yes (55 mmHg)	MVR	No	Myxoid degeneration
Hiromi Yano et al. (18)	M	61	Yes	A3	N/A	Yes (120 mmHg)	Morrow + MVR	No	N/A
Wakeyama et al. (19)	M	38	No	A3-P3	Yes (105 mmHg)	No	Medical therapy	N/A	N/A
Araujo et al. (20)	F	63	Yes	P3	Yes (103 mmHg)	No	MVR + TAP	N/A	Mucoid degeneration without inflammation
Koshino et al. (21)	F	63	Yes	P2	N/A	Yes (148 mmHg)	MVR	No	Myxoid degeneration
Morris and Ibrahim (22)	M	62	Yes	A2-P3	Yes (85 mmHg)	No	Morrow + MVR + Maze	No	Myxomatous degeneration of the mitral valve
Yang et al. (23)	F	69	Yes	P3	N/A	Yes (71 mmHg)	Morrow + MVr	N/A	N/A
Halpern et al. (10)	F	NA	N/A	PML	Yes (35 mmHg)	Yes (45 mmHg)	Medication	N/A	N/A
Halpern et al. (10)	M	NA	N/A	P1	Yes (80 mmHg)	No	Morrow + MVR	No	Myxomatous degeneration of PML and its chordae, similar to Barlow’s disease
Halpern et al. (10)	M	NA	N/A	P2	Yes (42 mmHg)	No	Morrow + MVR	No	Myxomatous degeneration of PML and its chordae, similar to Barlow’s disease
Halpern et al. (10)	M	NA	N/A	P2	Yes (67 mmHg)	No	Morrow + MVr	No	Myxomatous degeneration of PML and its chordae, similar to Barlow’s disease
Halpern et al. (10)	M	NA	N/A	P2-P3	Yes (60 mmHg)	No	Morrow + MVr	No	Myxomatous degeneration of PML and its chordae, similar to Barlow’s disease
Halpern et al. (10)	M	NA	N/A	P3	Yes (50 mmHg)	No	Morrow + MVr	No	Myxomatous degeneration of PML and its chordae, similar to Barlow’s disease
Suehiro et al. (24)	F	76	Yes	P2	Yes	Yes (76 mmHg)	Mitraclip	Yes (36 mmHg)	N/A
Wong et al. (25)	F	78	Yes	P3	Yes (102 mmHg)	No	Medication	N/A	N/A
Ieki et al. (26)	F	78	Yes	P2	Yes (100 mmHg)	Yes (49 mmHg)	MVr	N/A	N/A

AML, anterior mitral leaflet; PML, posterior mitral leaflet; Morrow, Morrow’s operation; MVr, mitral valve repair; MVR, mitral valve replacement; MAZE, MAZE operation; TAP, tricuspid annuloplasty; LVOTO, left ventricle outflow tract obstruction; AD-HF, admission for heart failure; N/A, not applicable.

In different series of patients, histological examination of the mitral chordae lesion revealed mucoid or myxomatous degeneration without inflammation (11, 13, 18, 21–23). The first explanation given for this structural alteration includes the combination of high LV systolic pressure and anterior displacement of the chordae tendineae which pull the AML toward the ventricular septum during the high-velocity blood jet ejection through the anatomically narrowed LVOT. This may generate a continuous fiber stretching and it may lead to progressive elongation of the leaflets, mucous degeneration, and chordal rupture (28). In this way elongation could be an adaptative process induced by the recurrence of AML-SAM into the narrowed LVOT, causing an increased endothelial shear rate that reactivates embryonic growth pathways (29). Although fascinating, this theory was confuted by two big bugs. First of all, even if the “Venturi effect” regards exclusively the AML, the entire valve presents histological metaplasia and chordae/leaflets elongation. In addition, chordae tendineae rupture occurs more frequently at the PML than at the anterior one (11, 21). Moreover, elongation of MV leaflets has been noticed in patients carrying gene mutations related to HCM but who have not yet the phenotypic expression of LV hypertrophy, suggesting that elongation might be a *primary phenotypic expression* of HCM (30). In the study published by Maron et al. (8), it was found that there was no relationship between AML or PML length and LV mass index or septum thickness. On the contrary, an association was found between mild hypertrophy and markedly elongated mitral leaflets (>30 mm in length) compared with patients with extreme LV hypertrophy. Groarke et al. (9) demonstrated that subjects found to be carriers of HCM sarcomere mutations have MV leaflets disproportionately longer compared with their LV cavity size. However, literature reports several controversies about the prevalence of myxomatous leaflet degeneration, similar to that observed in Barlow’s disease, in HCM. In a large anatomical study of patients with obstructive HCM, there was no histopathological myxomatous change in the *spongiosa* of excised MVs (31). The currently most accredited theory about the flail occurrence in HCM is that in patients with a genetically induced chordae/leaflets elongation, the traction induced by the drag forces through the narrowed LVOT may provoke the chordae rupture by itself (11). In fact, before rupture, almost all patients present mitral SAM and significant LVOT resting obstruction, which disappears in some cases after the flail appearance (11–13, 15, 20, 21, 23, 26). Decrease or disappearance of mitral SAM and LVOT obstruction after chordal rupture highlights the potential role of the sub-valvular apparatus as a cause of obstruction in HCM, while ventricular dilatation, due to significant MR, may expand the outflow tract reducing the drag forces. Hence, in cases like these, the reduction of the LVOT gradient may partly explain the paucity of symptoms in some patients, despite a severe MR appearance (11). The rupture of chordae tendineae in patients with narrowed LVOT can cause a lack of support of the AML, shifting the coaptation more posteriorly with the resolution of SAM and LVOT obstruction (26). Chordal rupture in HCM is rare and occurs more often in male-aged patients with obstructive disease and more frequently involves PML than AML (11, 12, 26). Although both AML and PML are elongated in HCM, PML elongation is usually less important than AML elongation, suggesting that insufficient elongation of PML could promote rupture, while the significant elongation of the AML might prevent chordal rupture (11).

#### Hemodynamic and clinical impact

Chordal rupture in HCM has been noticed to cause two major, opposite, hemodynamic changes with two opposite clinical manifestations. One is the negative effect of acute and severe MR, which affected first reported cases in the literature, with rapid hemodynamic deterioration due to acute/severe MV dysfunction, requiring urgent surgical treatment. The other one is the positive effect due to LVOT obstruction relieving with the achievement of clinical stability and reduced exertional dyspnea despite severe MR and abnormal LV wall thicknesses (21, 26).

An explanation of these different effects may be related to the degree of MR before the chordal rupture. In other words, if a severe MR due to AML-SAM is already present before the chordal rupture, it is possible that HF symptoms improve due to the resolution of LVOT obstruction alone while the severity of MR does not change. The findings of previous cases and of the present case indicate that clinical manifestations of chordal rupture in obstructive HCM vary depending on the degree of alleviation of LVOT obstruction and the degree of emerging acute MR. If chordal rupture leads to a torrential MR in a patient with previous mild/moderate SAM-related MR, it is possible that this type of complication may cause a hemodynamic deterioration.

#### Echocardiographic and Doppler features

**FIGURE 4:**
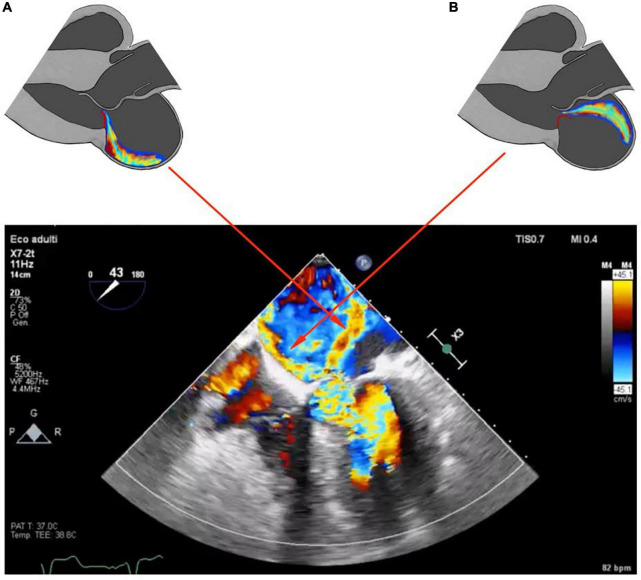
Schematic representation of parasternal long axis view showing two different types of mitral regurgitation jets, SAM-related and flail-related. Panel **(A)** shows the posteriorly directed jet in SAM-related MR. Panel **(B)** shows the anteriorly directed jet of MR in PML-flail. In red the different motions of PML related to the two different jets. Panel **(C)** shows the two different color jets found in the reported case; red arrows correlate the two color jets with the respective schematic representation.

Systolic anterior motion-related MR has been characterized by color Doppler echocardiography by a regurgitant jet directed posteriorly, occurring during the ejection phase of the systole. A central or anteriorly directed MR jet has been otherwise deemed to be diagnostic of intrinsic MV involvement. In particular, even if usually an MR originating from PML-flail has an anterior direction, in a patient with obstructive HCM it is often directed centrally because of the concomitant double component of PML-flail and AML-SAM, making harder the comprehension of the true mechanism (32). Figures 4A,B show this double mechanism. Another peculiarity of chordae rupture in obstructive HCM patients is that it can paradoxically resolve LVOT obstruction and SAM. In fact, the abnormalities in the mitral sub-valvular apparatus, such as hypertrophied or anteriorly displaced papillary muscle or apical-basal muscle bundles, displace the MV coaptation point anteriorly, closer to the septum. The thickened septum causes the flow drag to push MV anteriorly, resulting in SAM and LVOT obstruction. The rupture of chordae tendineae shifts posteriorly to the coaptation point by causing a lack of support to the AML with the resolution of SAM and LVOT obstruction (16). So it is not unusual to find that a worse, centrally directed, MR is accompanied by a wider LVOT, reduced LVOT gradient, and SAM disappearance in a patient previously known for an obstructive form of HCM (21).

Concomitant primary disease of the MV may lead to a different clinical decision-making process in patients with HCM undergoing surgical intervention, including the addition of MV repair/replacement (32). In our patient septal myectomy alone might not have completely eliminated AML-SAM. Neochords implantation could have contributed to achieving this result by shifting the coaptation line posteriorly.

## Conclusion

In obstructive HCM, mitral chordal rupture is a rare but serious complication that could change the clinical presentation in two different and opposite ways. When the LVOT obstruction drives the syntoms chordal rupture causes relief and, on contrary, when SAM and mitral regurgitation are prevalent it precipitates the hemodynamics. In the present case, heart failure symptoms and LVOT obstruction were not resolved after the rupture of MV chordae. Pre-operative echocardiography (both TTE and 2D-3D TEE) can demonstrate MV abnormalities in patients with HCM. These patients should never be treated by septal reduction alone. Surgical MV repair or replacement, in association with septal myectomy, is the preferred approach (33).

## Author contributions

MM and AS: manuscript writing. GG and SD’A: literature searching and references setting. UI: table, figures, and video setting. VP: supervisor. All authors contributed to the article and approved the submitted version.

## Supplementary material

Supplementary Video 1 Two-dimensional trans-esophageal echocardiogram, 4C view demonstrating: (1) severe hypertrophy of basal interventricular septum; (2) AML-SAM; and (3) mitral chordal rupture and PML-flail.

Supplementary Video 2 Two-dimensional trans-esophageal echocardiogram, mid-esophageal 3C-view (143°) demonstrating: (1) LV basal interventricular septum obstructive hypertrophy; (2) AML-SAM; and (3) PML-flail from chordal rupture.

Supplementary Video 3 Two-dimensional trans-esophageal echocardiogram, mid esophageal 5C-view (43°) demonstrating: (1) severe hypertrophy of basal i.v. septum; (2) AML-SAM; (3) chordal rupture + PML-flail; and (4) normal-sized LV with normal EF (%).

Supplementary Video 4 Two-dimensional trans-esophageal echocardiogram, mid esophageal 4C-view (43°), color Doppler assessment, demonstrating: (1) one MR anteriorly directed jet, related to PML-flail; and (2) one MR posteriorly directed jet, related to AML-SAM.
